# Immunogenicity of Virus-like Particles Based on VP1 Protein of Bovine Norovirus

**DOI:** 10.3390/vetsci12090802

**Published:** 2025-08-24

**Authors:** Zhigang Ma, Xuelian Ma, Xinyu Tao, Yong Huang, Qian Jiang, Xiaojun Ding, Fang Min, Yichen Chu, Ru Li, Xinying Zhang, Lu Liu, Caiyun Zhang, Qi Zhong, Gang Yao

**Affiliations:** 1 College of Veterinary Medicine, Xinjiang Agricultural University, Urumuqi 830052, China; mzg4334@nwafu.edu.cn (Z.M.); 2 Xinjiang Key Laboratory of New Drug Research and Development for Herbivores, Urumqi 830052, China; 3 College of Veterinary Medicine, Northwest A&F University, Yangling, Xianyang 712100, China; 4 Tacheng Vocational and Technical College, Tacheng 843300, China; 5 Xinjiang Uygur Autonomous Region Animal Husbandry and Veterinary Society, Urumuqi 830011, China

**Keywords:** bovine norovirus, virus-like particles, immunogenicity

## Abstract

Bovine Norovirus (BNoV) is a member of the enterovirus family that can cause gastroenteritis in calves. This virus poses a significant risk to calf growth and development as well as to the long-term sustainability of the cattle industry in China and elsewhere. No specific treatment or vaccine is currently available; thus, the development of a safe and effective vaccine is paramount. Here, we describe a strategy to assemble BNoV virus-like particles (VLPs) using the insect baculovirus expression system (BEV) to express the major structural protein, VP1, and demonstrate their potentiality as vaccines. The results showed that the BNoV-VLP self-assembled into complete spherical particles with a diameter of approximately 40 nm. When it was immunized in mice, the levels of specific IgG and IgA antibodies peaked at weeks 6 and 7 post-immunization, respectively, with maximum titers of 1:25,600 and 1:200. Moreover, we observed a significant increase in the CD4^+^/CD8^+^ T-cell ratio in splenic lymphocytes of immunized mice (*p* < 0.05), accompanied by a significant increase in TNF-α^+^CD4^+^ T-cells and TNF-α^+^CD8^+^ T-cells (*p* < 0.05). These results demonstrate that BNoV-VLPs are promising vaccine candidates for providing immunoprotection in the future. These studies support the significant practical implications of using a scientific basis for the development of a BNoV-VLP vaccine.

## Simple Summary

Bovine Norovirus (BNoV) virus-like particles (VLPs) were produced in an insect cell system to molecularly mimic the virus’s major structural protein, VP1. These VLPs efficiently self-assembled into spherical particles approximately 40 nm in diameter. When tested as a potential vaccine in mice, they elicited robust immune responses. Serum antibody titers peaked at weeks 6–7 post-immunization, reaching significant endpoint titers (IgG: 1:25,600; IgA: 1:200). Critically, the VLP also activated cellular immunity, significantly increasing the splenic CD4^+^/CD8^+^ T-cell ratio and enhancing the frequency of TNF-α-producing CD4^+^ and CD8^+^ T-cells following in vitro stimulation with BNoV-VLPs. These results demonstrate the potent immunogenicity of BNoV-VLPs, effectively inducing both humoral and T-cell-mediated immunity in mice. This comprehensive immunogenicity profile supports the further development of VLPs as a promising vaccine candidate for protection against BNoV infection in calves.

## 1. Introduction

Calf diarrhea is a significant cause of cattle mortality and economic losses in the livestock industry. The breakdown of intestinal ions, water, and nutrient transport in infectious diarrhea affects the growth and development of calves [1]. When calves are monitored for diarrhea symptoms, the focus is mainly on the culling procedure [2].

In recent years, the impact of BNoV on the cattle breeding industry has gradually attracted attention. BNoV was initially identified as an enterovirus responsible for bovine diarrhea in 1984 and is a single-stranded positive-stranded RNA virus belonging to the genus Norovirus in the Cuprioviridae family [3]. Calves infected with BNoV exhibited symptoms of diarrhea and lethargy, and in more severe instances, both watery diarrhea and intestinal lesions became apparent. These lesions are characterized by intestinal villus atrophy and damage to the epithelial cells of the intestinal villi [4]. Additionally, viral RNA was detectable in both fecal and blood samples during the period of infection with BNoV [4,5]. This shows that BNoV has a strong reproductive ability in vivo and can use different cell environments to maintain its survival and proliferation. Epidemiological survey results indicate the prevalence of BNoV worldwide. Cases of BNoV infection have been reported in numerous countries across continents, except for Antarctica. Countries such as Germany [6], Netherlands [7], the United States [8], South Korea [9], Belgium [10], Italy [11,12], Argentina [13], Egypt [14], Iran [15], Uruguay [16], and Australia [17] have recorded relatively high detection rates of BNoV, ranging from 7.5% to 61.67%. This has caused serious economic losses in these cattle industries. The emergence of BNoV is also a concern in the Chinese cattle trade. It is notable that China first tested for BNoV in 2017 [18]. Since then, BNoV has been reported in Xinjiang, Yunnan, Shanxi, Liaoning, Neimenggu, Henan, Hebei, and other provinces. However, effective preventive and therapeutic measures are currently lacking, resulting in a continued impact on the cattle industry [19,20,21].

Vaccination is often considered the most cost-effective method for preventing the transmission of contagious diseases. Common vaccine classifications include live attenuated vaccines, inactivated vaccines, subunit vaccines, DNA vaccines, and mRNA vaccines [22]. The virus-like particles (VLPs) vaccine is a subunit vaccine. At present, research on norovirus vaccines primarily focuses on developing VLP vaccines, and human norovirus (HuNoV)-based VLPs have entered the clinical trial stage [23]. VLPs are very similar to viruses in morphology and consist of viral structural proteins. These particles possess the structural characteristics of viruses, making them suitable for vaccine development, and VLPs share many characteristics with traditional vaccines but lack viral nucleic acids and do not replicate in the body, making them safer [24,25]. Studies have indicated that VLPs can be developed based on the BNoV VP1 protein [26]. Therefore, the development of BNoV vaccines based on VLPs is imperative and practicable for preventing calves from being infected with BNoV.

In conclusion, BNoV represents a global epidemic trend that has infected numerous provinces in China, resulting in significant economic losses to the cattle industry and posing a potential risk of zoonotic diseases [1]. Additionally, the absence of a BNoV vaccine and the inherent safety and efficacy advantages of VLPs, which mimic a natural pathogen without containing pathogenic genetic material, emphasize the pressing need for the development of a BNoV-VLP vaccine. Consequently, the objective of this study was to prepare BNoV-VLPs based on the VP1 gene of BNoV using BEVs and to evaluate their immunogenicity. This study will provide a valuable reference for the development of BNoV vaccines.

## 2. Materials and Methods

### 2.1. Gene and Plasmid Constructs

The reference sequence for the vaccine construct was derived from the VP1 gene of the Bo/XJ-KS/02/CHN strain (GenBank No. OM992315.1), which represents the prevalent GIII.2 genotype. The sequence had an Xho Ⅰ endonuclease site inserted at the 5′ end and a Hand Ⅲ endonuclease cleavage site and a 6 × His tag at the 3′ end. The VP1 gene of codon optimization was synthesized and ligated into the pFastBac1 vector to create the recombinant pFastBac1-VP1 transposable plasmid [27]. This plasmid was then transformed into DH10Bac receptor cells, and the VP1 gene was transposed into the Bacmid using the Bac-to-Bac system, resulting in the construction of the Bacmid-VP1 recombinant plasmid.

### 2.2. Cells, Animals, and Antibodies

The sf9 cells were purchased from the Chinese Typical Culture Preservation Center of Wuhan University. The sf9 cells began to proliferate 24 h after seeding, and once the cells reached over 90% confluency, they were passaged. The cells were collected by centrifugation at 1000× *g* for 10 min, and the supernatant was discarded. The cells were then passaged at a density of 2–3 × 10^5^/mL into new flasks containing 5 mL of complete medium (89% TNM-FH + 10% FBS + 1% penicillin–streptomycin). The cells were cultured at 28 °C without CO_2_.

SPF-BALB/C mice aged 4–6 weeks were purchased from the Animal Management Center at Xinjiang Medical University. The mice were allowed to acclimate to the new environment for 1–2 weeks before the experiment was conducted and had access to food and water ad libitum. The cages and their fittings were cleaned regularly, and after the experiment was completed, the mice were humanely euthanized.

The bovine anti-BNoV polyclonal antibody used in this study came from our laboratory and was stored at −20 °C. The His-Tag Mouse Monoclonal Antibody (HRP-conjugated) for Western blotting (WB) was purchased from Beyotime (Beyotime Institute of Biotechnology, Shanghai, China). The YSFluor488 goat anti-bovine IgG (H + L) antibody for indirect immunofluorescence was purchased from Yeasen (Yeasen Biotech Co., Ltd., Shanghai, China). The goat anti-mouse IgG H + L (HRP) and goat anti-mouse IgA H + L (HRP) for enzyme-linked immunosorbent assay (ELISA) were procured from Proteintech (Proteintech Group, Inc., Wuhan, China). FITC Anti-Mouse CD3, PerCP/Cyanine5.5 Anti-Mouse CD4, APC Anti-Mouse CD8a, FITC Anti-Mouse IFN-γ, and PE anti-mouse TNF-α for flow cytometry were purchased from BioLegend (BioLegend, Inc., San Diego, CA, USA).

### 2.3. Rescue of Baculoviruses and Production of BNoV-VLPs

Next, 80% of the cell amount was inoculated onto the plate according to the size of the flask. For each 6-well plate, approximately 1 × 10^6^ sf9 cells were inoculated. The cells were cultured in a medium at a constant temperature of 28 °C. After 16 h, the cells were adhered to the wall before transfection. Diluted Bacmid-VP1 was added gently to the diluted LipoInsect transfection reagent (Beyotime, China). The mixture was then gently mixed by tube inversion or pipetting and incubated at room temperature for 20 min. Next, 200 μL of the mixture of LipoInsect transfection reagent and Bacmid was uniformly added dropwise to each well. The control group comprised normal cells with LipoInsect transfection reagent. After 5 h, the culture medium was replaced with a complete culture medium, and the cells were incubated at 28 °C.

After approximately 96 h, when more than 80% of the sf9 cells appeared swollen, floating, stopped dividing, and underwent lysis, the supernatant was collected. The cells and cell debris were then precipitated by centrifugation for 5 min at 5000× *g* at 4 °C, and the supernatant was collected to be the P1. It was then preserved in sterile centrifuge tubes. To increase the virulence of the P1, it is necessary to amplify it further. Therefore, 200 μL of the collected P1 was taken to infect normal sf9 cells, which had been spread to T25 flasks in advance. The cell density should be about 80%. Next, the cells were cultured for 96 h at 28 °C. After more than 80% of the cells showed cytopathic lesions, the supernatant was collected by centrifugation to obtain the P2 baculovirus, which was then stored frozen at −80 °C. For inoculation, 8 mL of P2 baculovirus was added to a volume of 200 mL with a cell density of 3 × 10^6^ cells/mL. The culture was maintained at 28 °C and 135 rpm/min for 96 h without CO_2_, after which the culture supernatant was collected for purification.

### 2.4. SDS-PAGE and WB Verified the Expression of VLPs

The sample volume was calculated based on the protein quantification results. Then, the electrophoresis buffer was added and the sample was uploaded. Subsequently, the protein electrophoresis was run at a constant voltage until the dye front reached the bottom of the gel. Finally, an appropriate amount of Coomassie Brilliant Blue staining solution was added to stain the PAGE gel, followed by staining and decolorization.

The gel containing the target proteins was separated using SDS-PAGE. Subsequently, the separated proteins were transferred from the gel to a nitrocellulose membrane. The membrane was then blocked with blocking buffer (5% milk in TBST) for 2 h at room temperature to prevent nonspecific binding. Next, His Tag Mouse Monoclonal Antibody (HRP-conjugated) was incubated overnight at 4 °C. The nitrocellulose membrane was washed 5 times with TBST for 5 min between each step. Finally, the target proteins were visualized using the ECL luminescence method in a light-avoiding environment.

### 2.5. Indirect Immunofluorescence Verified the Expression of VLPs

The sf9 cells were seeded into 24-well plates containing coverslips at a density of 2–3 × 10^5^/mL. The cells were then incubated at 28 °C until they reached 80% confluency on the following day. P2 baculovirus was added at this point. The 24-well plates were collected after the cytopathic effect (CPE) appeared in the cells, which typically occurred within 24–36 h. The cells were fixed with 4% paraformaldehyde fixative at room temperature for 20 min. The cells were then treated with 5% Triton X-100 at room temperature for 30 min. After incubating the 1:500 dilution of bovine anti-BNoV polyclonal antibody overnight at 4 °C, a 1:500 dilution of YSFluor488 goat anti-bovine IgG (H + L) secondary antibody was incubated at room temperature for 1 h. The 0.5 μg/mL DAPI solution was incubated at room temperature for 10 min. Between each step, the plate was washed with PBS 3 times for 5 min. Subsequently, it was observed under a laser confocal microscope at an excitation wavelength of 488 nm.

### 2.6. VLPs Purification

To prepare a saturated ammonium sulfate solution, 767 g of (NH_4_)_2_SO_4_ was added slowly to 1 L of distilled water while stirring. Then, the pH was changed to 7.0 with either ammonia or sulfuric acid. Ammonium sulfate crystals emerged after 1 day of static settling, indicating that the ammonium sulfate solution was supersaturated (4.1 mol/L, 25 °C), using the supernatant for purification. To remove impure protein from the medium supernatant, saturated ammonium sulfate was added slowly while stirring to final concentrations of 33%, 35%, 37%, and 40%. Next, a protease inhibitor cocktail (1 × final concentration) was added, and the solution was incubated at 4 °C overnight to precipitate the protein. The supernatant was discarded after centrifugation at 12,000× *g* for 30 min at 4 °C on the following day. The precipitate was retained and then dissolved in a small amount of PBS.

### 2.7. Transmission Electron Microscopy (TEM)

The samples before and after purification were filtered through a 0.22 μm membrane. Then, 1–2 μL of the sample was dropped onto a copper grid, stained with tungsten phosphate, and observed under an electron microscope to determine the morphology of the VLPs.

### 2.8. Mice Immunization

**Figure 1:**
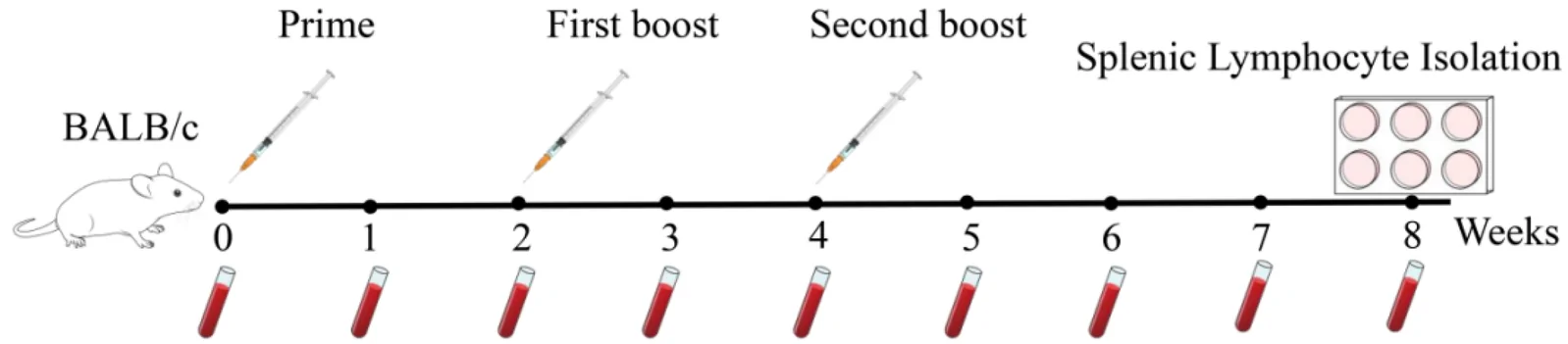
Immunization scheme.

The initial immunization consisted of 100 μg of antigen per mouse administered with Freund’s Complete Adjuvant. Booster immunizations were delivered in two doses with an inter-dose interval of 14 days. Freund’s incomplete adjuvant was selected for the booster immunizations, and 50 μg of antigen was administered to each mouse. At the same time, there was a control group that received approximately the same volume as the experimental group of PBS. And each group immunized 5 mice. Mice were immunized via subcutaneous injection at 6–8 sites (≤100 μL per site) with BNoV-VLPs emulsified in Freund’s Complete Adjuvant, with no observable adverse effects attributable to the immunization procedure or adjuvants. Blood samples were collected from all mice at various time points pre-immunization and post-immunization. Whole blood was obtained from mice by tail-cutting in 1.5 mL centrifuge tubes and kept at room temperature for 2 h, then centrifuged at 4 °C and 4000 g for 20 min. The serums were labeled and frozen at −20 °C. The specific immunization program is shown in Figure 1. During the experiment, the institutional animal ethics guidelines were strictly adhered to, and approval was obtained from the Institutional Animal Care and Use Committee (IACUC) under protocol number 2024054.

### 2.9. Enzyme-Linked Immunosorbent Assay

To determine the serum-specific IgG antibody levels against BNoV at different times, we used 1 μg/mL of the BNoV-VLPs diluted in coating buffer (pH = 9.6), and 100 μL of coating buffer was coated to each well of a 96-well plate and refrigerated at 4 °C overnight for coating. The next day, the plates were dried, and 200 μL of PBS-T containing 5% skimmed milk powder was added to each well and incubated at 37 °C for 2 h. After, 100 μL/well of mouse serum diluted by PBS (1:5000 dilution when detecting IgG; 1:500 dilution when detecting IgA) was added and incubated for 1 h at 37 °C. After incubation, 100 μL/well of goat anti-mouse IgG H&L (HRP) (1:50,000) or goat anti-mouse IgA H&L (HRP) (1:1000) was added and incubated for 1 h at 37 °C. The plate was washed 5 times with PBS-T for 5 min between each step. After incubation, 100 µL of TMB substrate solution was added to each well and incubated at 37 °C for 30 min. The reaction was then terminated by adding 50 µL of 2M H_2_SO_4_ to each well, causing the color to change from blue to yellow. The absorbance of each well value at 450 nm was measured using a microplate reader.

For antibody titer determination, mouse serum was subjected to serial dilution in PBS (ranging from 1:200 to 1:51,200) following established protocols. Subsequent procedures were carried out as previously described.

### 2.10. Splenic Lymphocyte Isolation

Following euthanasia of three mice per group, BALB/c mice were immersed in 75% ethanol for 5 min. Then, the spleens were extracted, and the external coat was removed, cut into small pieces (approximately half of each spleen), and filtered by grinding using a 70 μm cell filter, and rinsed with Hank’s buffer for collection. The cells were resuspended with precooled erythrocyte lysate and allowed to stand on ice for 5 min. The cells were washed with Hank’s buffer and collected by centrifugation at 500× *g*; afterward, they were resuspended in 2 mL of RPMI 1640 medium and diluted to 3 × 10^6^ cells/mL. Next, the cells were inoculated into 6-well plates and stimulated with BNoV-VLPs at a final concentration of 10 μg/mL, and finally cultured at 37 °C with 5% CO_2_.

### 2.11. Flow Cytometry

After culturing the splenic lymphocytes for approximately 42 h, the cells were collected from one well by centrifugation at 500× *g* for 5 min. Subsequently, the cells were suspended in 1 mL of PBS containing 5% FBS and treated with FITC Anti-Mouse CD3 antibody (5 μL/tube), PerCP/Cyanine5 Anti-Mouse CD3 antibody (5 μL/tube), and APC Anti-Mouse CD8a antibody. The cells were incubated for 20 min at room temperature, protected from light, and then washed once with PBS containing 1% bovine serum albumin (BSA). Subsequently, 100 μL of fixation buffer was added under light-protected conditions and incubated for 20 min at room temperature. After washing twice with PBS, the cells were resuspended in 200–500 μL of PBS and analyzed using a flow cytometer.

Protein transport inhibitors were added at a final concentration of 10 μg/mL after culturing the splenic lymphocytes for approximately 42 h. After 6 h, the cells were collected from one well by centrifugation at 500× *g* for 5 min. A tube of cells was incubated with 100 μL of fixation buffer and PerCP/Cyanine5.5 Anti-Mouse CD4 antibody (5 μL/tube) and APC Anti-Mouse CD8a antibody (5 μL/tube) for 30 min at room temperature and protected from light and then washed once with PBS. Next, 100 μL of permeabilization buffer, 5 μL/tube of FITC Anti-Mouse IFN-γ antibody, and 5 μL/tube of PE anti-mouse TNF-α antibody were added. These were mixed well and incubated for 30 min at room temperature under light protection. After incubation, the cells were washed twice with PBS. Finally, the cells were resuspended in 200–500 μL of PBS and analyzed using the flow cytometer.

### 2.12. Statistical Analysis

Each experimental group was subjected to three biological replicates and three technical replicates. Statistical analysis of intergroup data differences was performed using GraphPad Prism 8.2.1 software. Data for each group were visualized as grouped plots, with values presented as the mean ± standard error of the mean (SEM). A two-tailed unpaired Student’s *t*-test was employed for comparisons, where a *p*-value < 0.05 was considered statistically significant (*) and *p* < 0.01 was deemed highly statistically significant (**).

## 3. Results

### 3.1. Rescue of Baculoviruses

**Figure 2:**
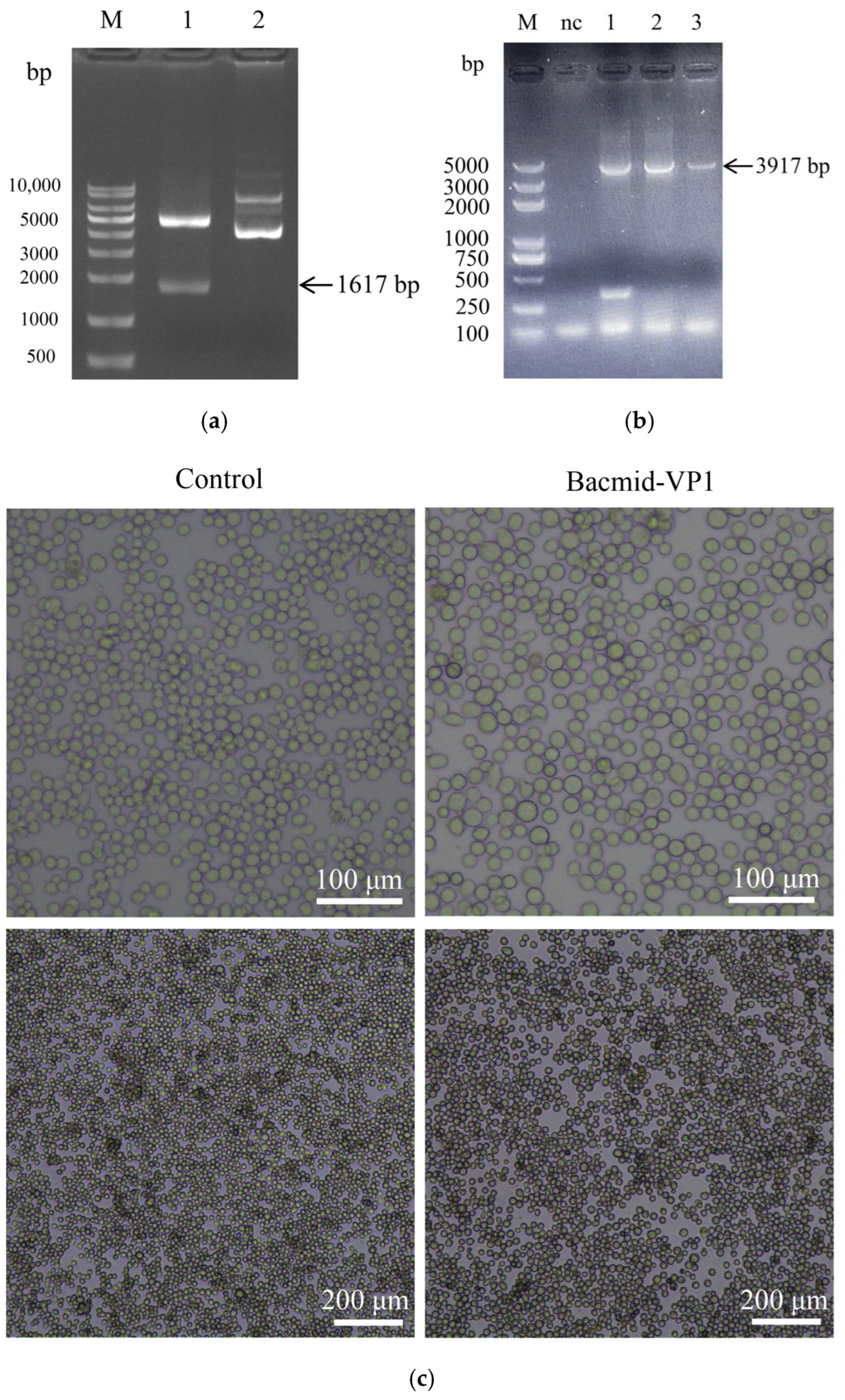
Results of rescue of baculoviruses. (**a**) Result of pFastBac1-VP1 dual-enzyme digestion, lane M: 10,000 bp DNA ladder; lane 1: pFastBac1-VP1 dual-enzyme digestion, and lane 2: pFastBac1 no dual-enzyme digestion. (**b**) Result of Bacmid-VP1 PCR, lane M: 5000 bp DNA ladder; lane nc: negative control and lanes 1–3: Bacmid-VP1. (**c**) Bacmid-VP1 was transfected into sf9 cells.

As shown, the recombinant plasmid pFastbac1-VP1 was constructed by using the codon-optimized sequence of the VP1 gene of the Bo/XJ-KS/02/CHN strain, and then double enzyme digestion verified that the fragment size was 1617 bp, indicating that it was correct (Figure 2a). Subsequently, the VP1 was transposed to the Bacmid, and the recombinant Bacmid-VP1 was constructed. The successful transposition fragment was verified by PCR and characterized by a fragment of 3917 bp (1617 + 2300 bp) (Figure 2b). The recombinant Bacmid-VP1 was transfected into sf9 cells. As illustrated, the transfected sf9 cells gradually exhibited characteristic signs of cytopathic effects (CPEs), such as cell enlargement, suspension, and reduction in cell density. Upon surpassing a predefined threshold where more than 80% of the cells displayed CPE, the cell culture was promptly terminated (Figure 2c).

### 3.2. Production and Purification of VP1 Proteins in sf9 Cells

**Figure 3:**
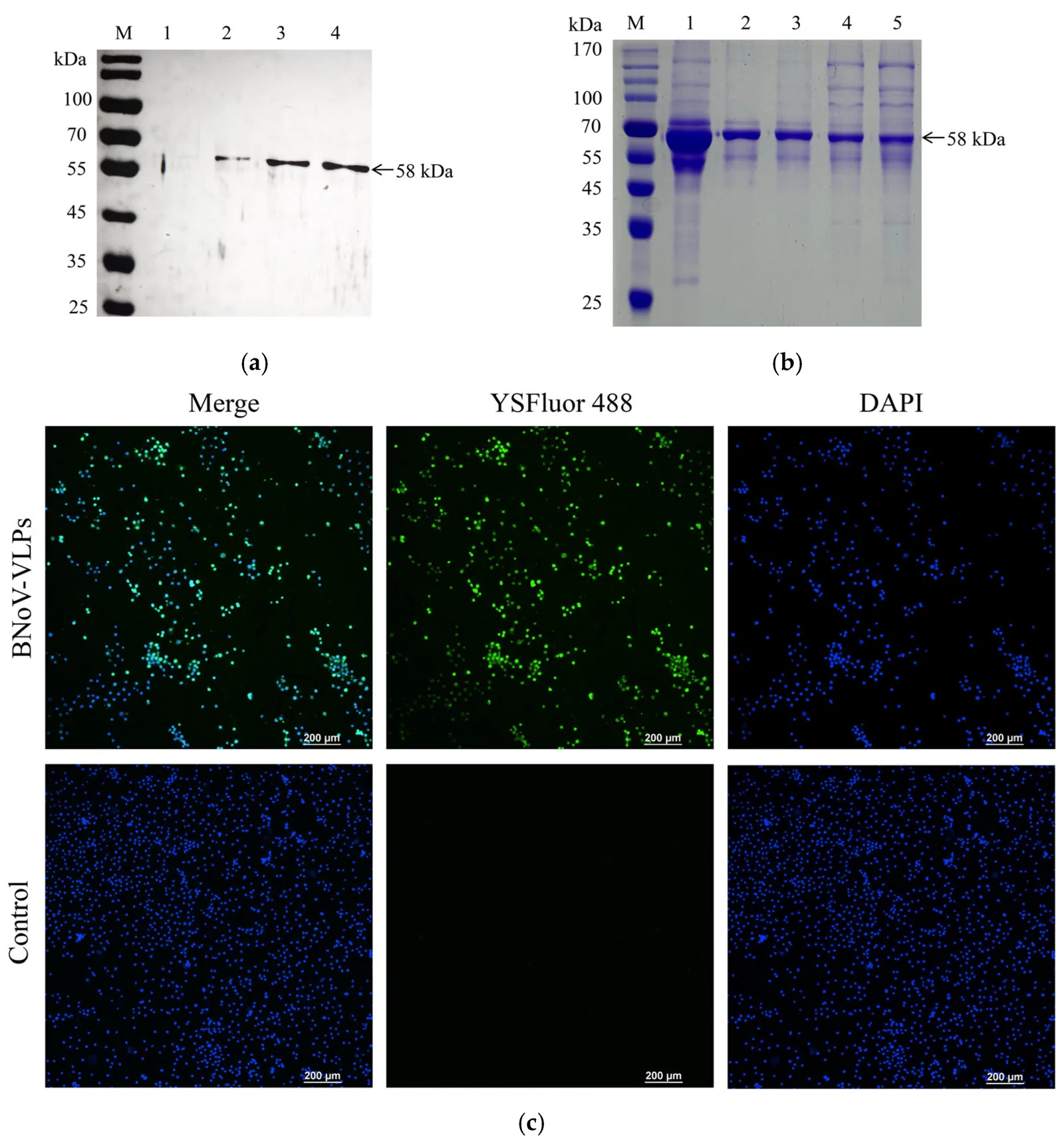
Production and purification of VP1 protein in sf9 cells. (**a**) The expression of the VP1 protein was verified by WB, Lane M: protein molecular weight marker (10–170 kDa), lane 1: untransfected Bacmid-VP1, and lanes 2–4: transfection of Bacmid-VP1. (**b**) The purification results of the BNoV-VLPs were verified by SDS-PAGE, Lane M: protein molecular weight marker (10–170 kDa), lane 1: crude BNov-VLPs, lane 2: BNov-VLPs purified by 33% ammonium sulfate precipitation, lane 3: BNov-VLPs purified by 35% ammonium sulfate precipitation, lane 4: BNov-VLPs purified by 37% ammonium sulfate precipitation, and lane 5: BNov-VLPs purified by 40% ammonium sulfate precipitation. (**c**) The expression of VP1 in sf9 cells was verified by indirect immunofluorescence.

After the initial round of P1 baculovirus expression, the supernatants from the cells were harvested. As depicted in Figure 3a, a specific band at 58 kDa was observed in the supernatants from the sf9 cell medium after transfection with Bacmid-VP1. This band was not present in nontransfected sf9 cells, indicating successful VP1 protein expression confirmed by WB analysis.

To further validate the VP1 protein expression level, sf9 cells were examined for 24 h to 36 h after P1 baculovirus infection. The results showed that Bacmid-VP1-transfected sf9 cells appeared fluorescent green (Figure 3c), whereas nontransfected sf9 cells did not appear fluorescent green, confirming successful VP1 protein expression in the sf9 cells.

We used the nickel affinity chromatography column purification method. However, due to structural constraints, the His tag was not exposed on the protein surface, making the BNoV-VLPs unable to combine with the nickel affinity chromatography column. Therefore, this purification method is unsuitable for purifying BNoV-VLPs. Consequently, the experiment employed a more cost-effective and straightforward ammonium sulfate method to purify BNoV-VLPs. Saturated ammonium sulfate was added to the medium’s supernatant in varying ratios to adjust the ammonium sulfate concentration. As demonstrated, the purity of the BNoV-VLPs exceeded 95% at a 33% ammonium sulfate concentration (Figure 3b). Subsequent dialysis removed the residual target protein, yielding a high concentration of the target protein. Further dialysis was conducted to eliminate ammonium sulfate residue and concluded that the concentration of the purified BNoV-VLPs could reach 1.2 mg/mL by the BCA protein assay kit.

### 3.3. VLPs Morphology Analysis via TEM

**Figure 4:**
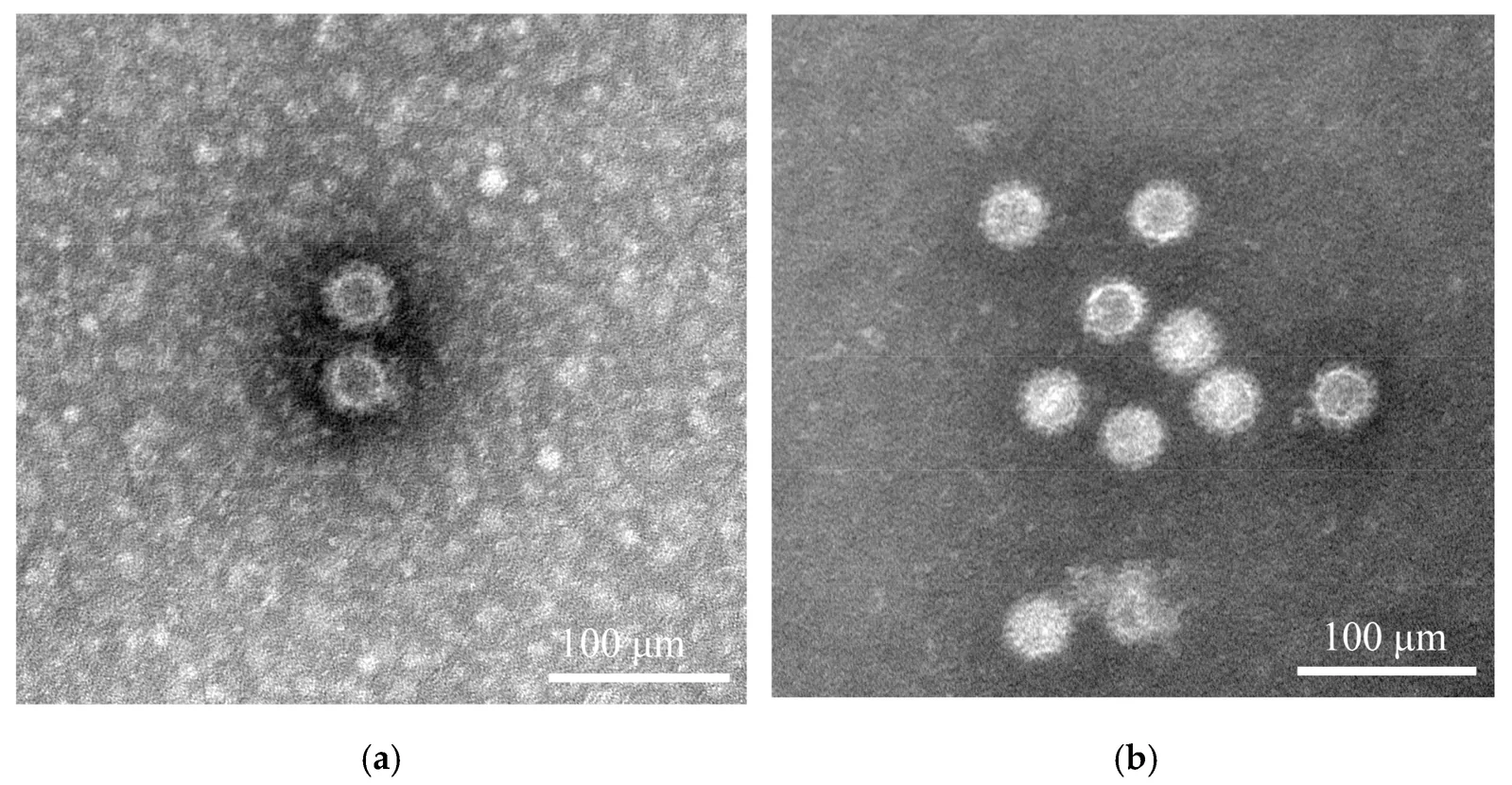
The assembly of BNoV-VLPs was verified by transmission electron microscopy. (**a**) Before purification of BNoV-VLPs. (**b**) After purification of BNoV-VLPs.

The structural characteristics of the BNoV-VLPs were assessed using transmission electron microscopy. As depicted, both prepurification and postpurification BNoV-VLPs were characterized as particles approximately 40 nm in size, which closely mirrors that of natural BNoV particles (Figure 4). This indicates the ability of the VP1 protein to self-assemble into BNoV-VLPs within sf9 cells. Moreover, the structural characteristics of the BNoV-VLPs remained intact after purification.

### 3.4. IgG Antibody Levels Against BNoV

**Figure 5:**
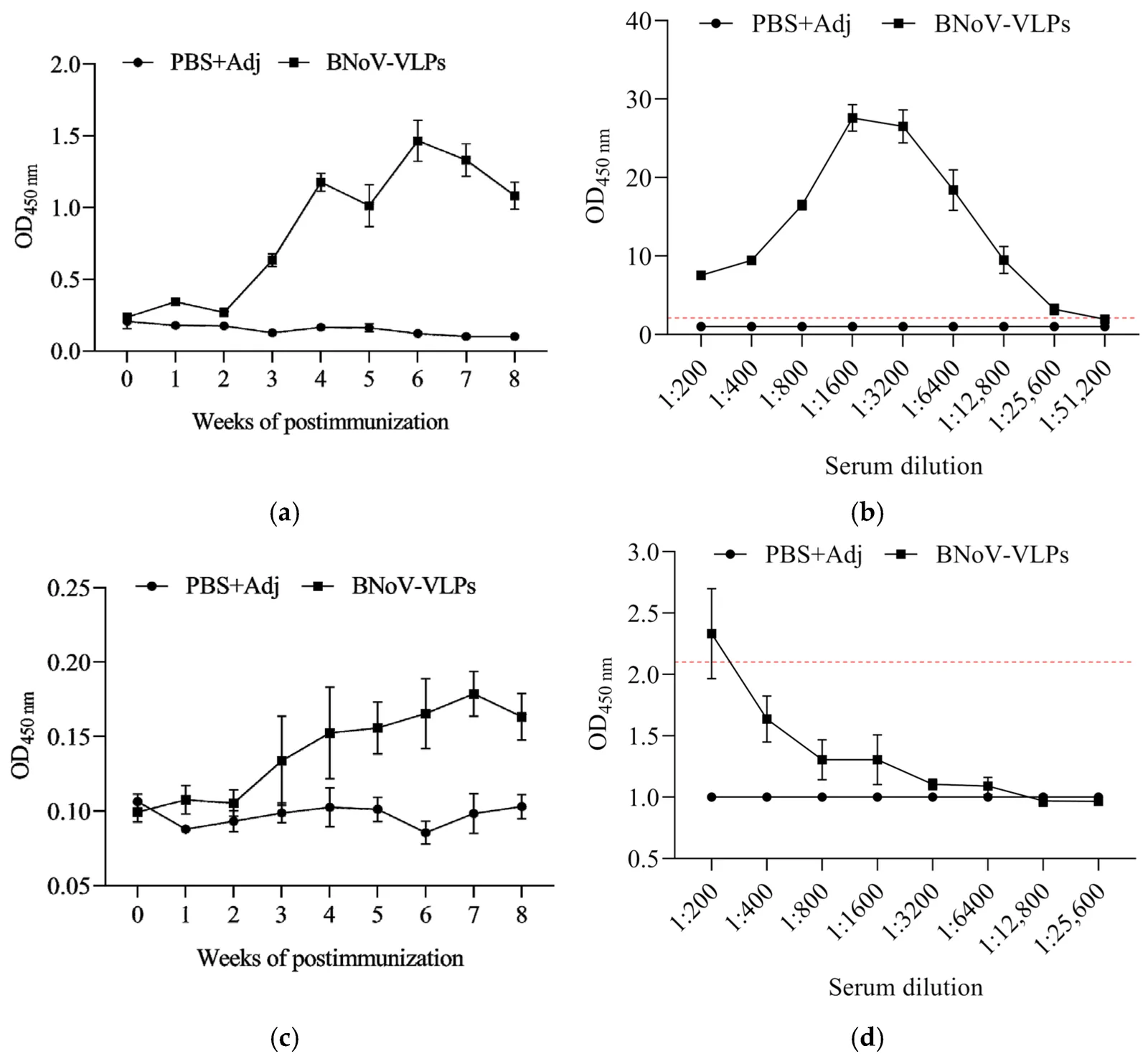
Antibody levels against BNoV. (**a**) IgG antibody levels in different periods at 1:5000 dilution; (**b**) IgG antibody titer; (**c**) IgA antibody levels in different periods at 1:500 dilution; (**d**) IgA antibody titer. Data represent mean ± SD of individual mouse sera (*n* = 5 per group).

To monitor changes in the humoral immune response triggered by BNoV-VLPs, we diluted mouse serum collected from 0 to 8 weeks pre-immunization at a ratio of 1:5000 using indirect ELISA and tracked variations in specific IgG antibody levels at different time points. As depicted, the experimental group exhibited a distinct binding affinity to BNoV-VLPs compared to the control group (Figure 5a). The IgG antibody levels against BNoV in the experimental and control groups were similar. However, from the first week of post-immunization, there was a gradual increase in IgG antibody levels, with a slight decrease observed in the second week, followed by a more pronounced rise from the second to the fourth week. Subsequently, there was a decline in antibody levels during the fifth week, followed by a peak in the sixth week (second week after the second booster immunization), before gradually decreasing in the seventh week. IgG antibody levels in the eighth week resembled those observed during the fourth to fifth weeks.

To evaluate the titer of serum-specific IgG antibodies against BNoV, the maximum dilution at which serum can still bind to the VLPs was determined. Serum collected at week 6 post-immunization was diluted at various ratios (1:200, 1:400, 1:800, 1:1600, 1:3200, 1:6400, 1:12,800, 1:25,600, and 1:51,200). A sample was considered positive and the antibodies thus produced were deemed effective when the absorbance value of the BNoV-VLP group divided by that of the PBS + Adj group exceeded 2.1. Results showed that the experimental group exhibited a significant difference from the control group, and maximum antibody potency was observed at a dilution of 1:25,600, according to this criterion (Figure 5b). This indicates that BNoV-VLPs can stimulate the production of elevated levels of IgG antibody titers, thereby enhancing the humoral immune response.

### 3.5. IgA Antibody Levels Against BNoV

We diluted the mouse serum collected from 0 to 8 weeks at a ratio of 1:500 using an indirect ELISA. Tracked variations in specific IgA antibody levels at different time points allowed us to measure the levels of specific IgA antibodies at various time points. The immunization group showed specific binding to BNoV-VLPs compared to the control group. Pre-immunization, IgA antibody levels against BNoV in both groups were equivalent (Figure 5c). Post-immunization, IgA antibody levels began to rise in the first week, experienced a slight dip in the second week, and peaked in the seventh week (three weeks after the second boost immunization). Subsequently, levels gradually declined by the eighth week.

Therefore, the serum-specific IgA antibody titer represents the maximum serum dilution capable of binding to BNoV-VLPs at the seventh week using an indirect ELISA. Serum collected at week 7 post-immunization was diluted at various ratios (1:200, 1:400, 1:800, 1:1600, 1:3200, 1:6400, 1:12,800, and 1:25,600). A sample was considered positive and the antibodies thus produced were deemed effective when the absorbance value of the BNoV-VLP group divided by that of the PBS + Adj group exceeded 2.1. The results demonstrated a significant difference between the immunization and control groups, and the maximum antibody titer was observed at 1:200, according to this criterion (Figure 5d). This indicates that BNoV-VLPs can stimulate the production of elevated levels of IgA antibody titers.

### 3.6. CD4^+^/CD8^+^ T-Cell Ratio

**Figure 6:**
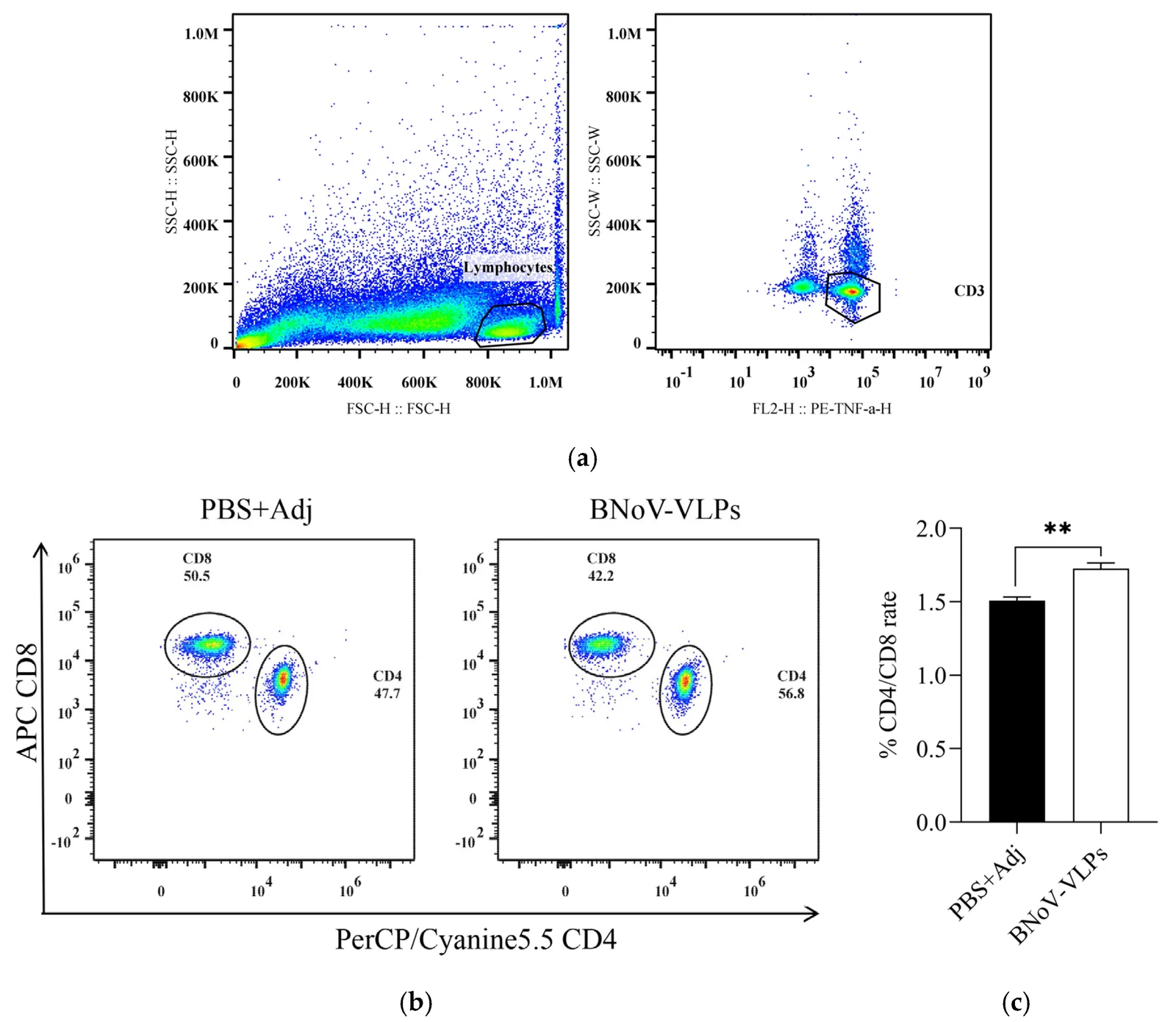
CD4^+^/CD8^+^ T-cells ratio after immunization. (**a**) Gating strategy; (**b**) CD4^+^ and CD8^+^ T-cells ratio; (**c**) frequency of CD4^+^/CD8^+^ T-cells. Data in (**b**,**c**) represent mean ± SD of splenic CD4^+^/CD8^+^ ratios from individual mice (*n* = 3); “**” in (**c**) means *p* < 0.01.

To assess the cellular immune response elicited by BNoV-VLPs, splenic lymphocytes were isolated from mice at the eighth week post-immunization. After being stimulated by BNoV-VLPs, the levels of CD4^+^/CD8^+^ T-cells were detected using flow cytometry (FCM). Gate 1 (representing lymphocytes) was delineated from total cells, Gate 2 (representing CD3^+^ T-cells) was delineated from Gate 1, and subsequently, CD4^+^/CD8^+^ T-cells were distinguished from Gate 2 (Figure 6a). This demonstrated that, compared with the control group, the values of CD4^+^/CD8^+^ T-cells in splenic lymphocytes of mice immunized in the eighth week exhibited a significant increase (*p* < 0.05) (Figure 6b,c). This result suggests that BNoV-VLPs could induce mice to produce T-cell immune responses, predominantly causing an increase in CD4^+^ T-cells and a decrease in CD8^+^ T-cells.

### 3.7. IFN-γ and TNF-α Levels

**Figure 7:**
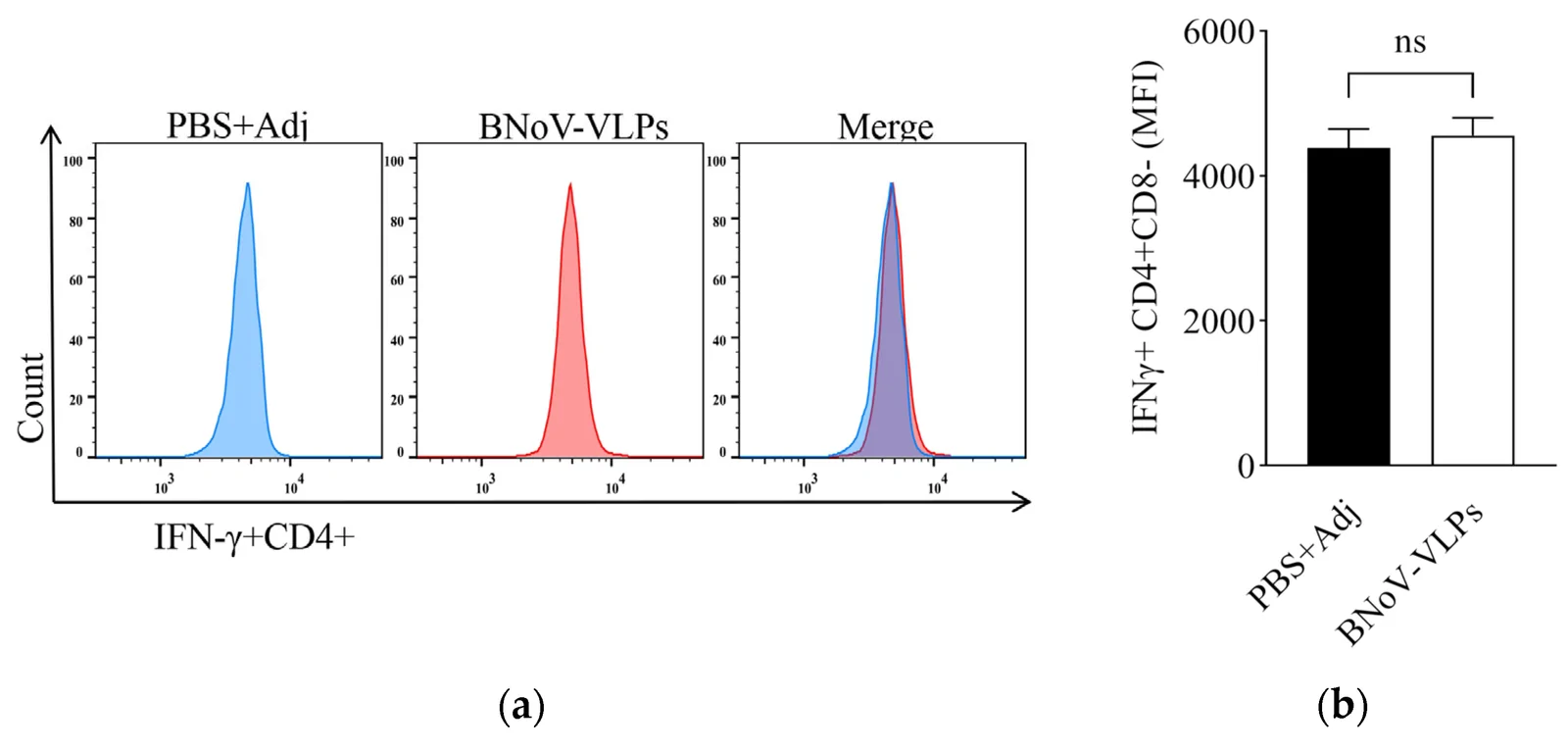

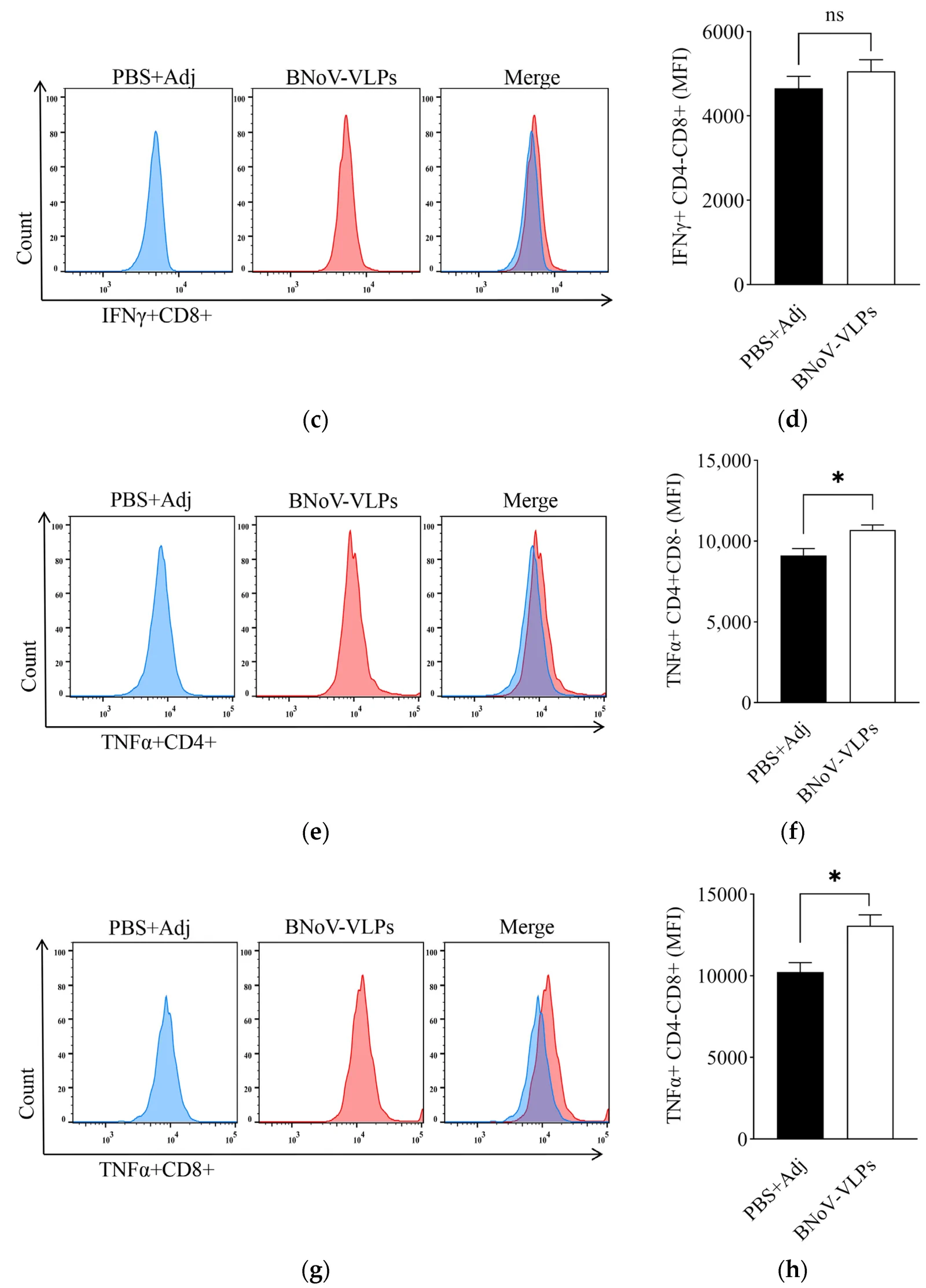
IFN-γ and TNF-α cytokine levels in cells. (**a**) IFN-γ in CD4^+^ T-cells by FCM. (**b**) Quantification of IFN-γ in CD4^+^ T-cells. (**c**) IFN-γ in CD8^+^ T-cells by FCM. (**d**) Quantification of IFN-γ in CD8^+^ T-cells. (**e**) TNF-α in CD4^+^ T-cells by FCM. (**f**) Quantification of TNF-α in CD4^+^ T-cells. (**g**) TNF-α in CD8^+^ T-cells by FCM. (**h**) Quantification of TNF-α in CD4^+^ T-cells. All quantifications (**b**,**d**,**f**,**h**) show mean ± SD of intracellular cytokine staining from individual mouse spleens (*n* = 3). “ns” in (**b**,**d**) means no statistical significance; “*” in (**f**,**h**) means *p* < 0.05.

Spleen lymphocytes were isolated and then stimulated by BNoV-VLPs. The levels of IFN-γ and TNF-α produced by T-cells post-immunized from the eighth week were scrutinized. It was observed that the IFN-γ^+^CD4^+^ and IFN-γ^+^CD8^+^ T-cells in mice immunized with BNoV-VLPs exhibited an increase compared to the control group, although it was not statistically significant (*p* > 0.05) (Figure 7a–d). However, the TNF-α^+^CD4^+^ and TNF-α^+^CD8^+^ T-cells displayed a significant increase (*p* < 0.05), suggesting that BNoV-VLPs predominantly stimulated mouse T-cells to produce TNF-α (Figure 7e–h).

## 4. Discussion

The cattle industry in China is facing a significant threat due to the pervasive presence of BNoV in the region, as highlighted by previous studies [20]. The absence of a commercially available vaccine against BNoV highlights the pressing need for the development of a safe and effective vaccine. Currently, some VLP vaccines have been commercialized, such as the porcine circovirus type 2 (PCV2) vaccine [28], hepatitis B virus (HBV) vaccine and hepatitis E virus (HEV) vaccine [29]. These findings have shown good immunogenicity and safety. The ability of VLPs to mimic pathogens without causing infection makes them an attractive platform for vaccine development. They can stimulate a robust immune response without posing a risk to the host. These advantages make VLPs a promising alternative to traditional vaccines, providing a novel and more advantageous vaccine strategy. BNoV-VLPs, comprising 180 VP1 monomeric proteins, have been shown to trigger a strong immune response in organisms, surpassing that induced by monomeric proteins [30]. Moreover, BNoV-VLPs have exhibited remarkable stability, remaining intact for extended periods at 4 °C and being capable of reassembly into larger-diameter VLPs under specific conditions following dissociation [30].

There are five commonly used expression systems for producing VLPs: the *E. coli* expression system [31], BEVs [32], the yeast expression system [33], the eukaryotic cell expression system [34], and the plant expression system [35]. BEVs were pioneered by Smith et al. in the early 1980s [36]. BEVs facilitate gene expression by infecting insect cells and producing recombinant proteins. To summarize, BEVs have several advantages. First, they exhibit enhanced gene expression. The polyhedron (ph) promoter and very late promoter (p10) in insect cells enhance the efficiency of exogenous gene expression and facilitate large-scale culture [37]. Furthermore, through post-translational modifications, BEVs are capable of selectively altering proteins to produce particles that closely resemble natural viruses in structure and function [38]. Furthermore, BEVs offer flexibility with exogenous fragments. They can accommodate insertions of up to 50 kb without interfering with gene expression and recombination, making it easy to add tags for protein purification later on [39]. These advantages make BEVs a versatile tool with a broad range of applications, including vaccine development, drug screening, and gene therapy. In our previous study, BEVs seemed to be a more promising option by codon preference analysis for BNoV VP1 protein production [27]. Therefore, in this study, we successfully prepared VLPs that closely resembled the natural structure of BNoV using BEVs. These VLPs had a uniform size of approximately 40 nm and were characterized by their intact, homogeneous spherical morphology (Figure 3), in agreement with prior reports of BNoV VLPs [10]. Furthermore, BNoV-VLPs can be combined with adjuvants to become candidates for vaccines.

Further, we immunized BALB/c mice with BNov-VLPS with Freund’s Adjuvant. Then, the antibodies (IgG and IgA) in the serum were evaluated during immunization. IgG antibodies play a crucial role in viral neutralization, eliciting an immune response and eliminating pathogens. As a result, they serve as vital indicators of vaccine immunogenicity. We then utilized indirect ELISA to quantify the levels of specific IgG antibodies in the serum samples. The results showed that IgG antibody titers increased significantly one week post-immunization with BNoV-VLPs compared to the control group (Figure 5). The peak IgG antibody titer was observed at six weeks, with a maximum antibody potency of 1:25,600. We noted a decrease in antibody titers at weeks 2, 5, and 7; however, this decline was reversed following booster immunization, which led to augmented production of IgG antibodies (Figure 5). In conclusion, we demonstrated that BNoV-VLPs can induce mice to produce elevated levels of serum-specific IgG antibodies with enhanced potency. Further research is needed to optimize the immunization protocol, including the selection of adjuvants, immunization schedule, and dosage.

IgA antibodies play a vital role in viral neutralization and the prevention of intracellular infection. Despite their relatively low abundance in serum compared to IgG antibodies, studies have demonstrated that IgA antibodies elicited by immunized VLPs exhibit enhanced viral neutralization capacity and preferential binding to specific epitopes on NoV particles. This confers superior protection against NoV infection [40,41]. Although secretory IgA (sIgA) functions as the primary effector at mucosal surfaces, its detection is hampered by significant technical challenges, including invasive sampling procedures (e.g., intestinal lavage). Given our translational objective to establish a standardized protocol for subsequent bovine trials where mucosal sampling is logistically impractical, serum IgA quantification provides a methodologically robust and clinically relevant metric for evaluating vaccine-induced immune responses in murine models prior to large-animal validation. In this study, we investigated the production of IgA antibodies following post-immunization with BNoV-VLPs. Our results revealed that elevated titers of specific IgA antibodies were detected one week post-immunization, with the peak titer observed at the seventh week reaching a maximum potency of 1:200. These findings suggest that immunization with BNoV-VLPs can stimulate the robust production of specific IgA antibodies, which may contribute to resistance to BNoV infection (Figure 5).

Research has demonstrated that VLPs stimulate macrophages, captured by antigen-presenting cells (APCs), and are transported through the major histocompatibility complex (MHC) class I molecular pathway to cross-present activated CD8^+^ T-cells [42]. Moreover, VLPs can be directly recognized by dendritic cells (DCs), promoting their migration and activation through the MHC class II molecular pathway to stimulate the production of CD4^+^ T-cells and trigger an immune system response [43,44,45,46,47]. Furthermore, studies have shown that HuNoV infection leads to a significant increase in CD4^+^ T-cells, accompanied by the production of TNF-α and IFN-γ. This study revealed that immunization with BNoV-VLPs led to a significant increase in the CD4^+^/CD8^+^ T-cell ratio within the splenic lymphocytes of mice at week 8 (*p* < 0.05).

However, the increase in IFN-γ^+^CD4^+^ T-cells and IFN-γ^+^CD8^+^ T-cells was not statistically significant (*p* > 0.05), likely because IFN-γ secretion peaks during the initial phase of infection [48]. Furthermore, the level of TNF-α^+^CD4^+^ T-cells and TNF-α^+^CD8^+^ T-cells was significantly elevated at week 8 (*p* < 0.05). As an inflammatory factor, TNF-α plays a crucial role in controlling pathogen infections and inducing apoptosis [49,50,51]. Studies have shown that TNF-α^+^CD4^+^ T-cells may be associated with neutralizing antibodies produced during antiviral resistance [52]. These results suggest that BNoV-VLPs may have the potential to influence the development of BNoV-induced diseases.

However, the development of such a vaccine is hindered by the absence of an in vitro culture system for BNoV and a reliable experimental animal model. This limitation has also made it difficult to establish reliable immune evaluation indices and immunoprotective effects. Given that host immune responses to murine norovirus (MNoV) and human norovirus (HuNoV) have been partially characterized, studies on Bovine Norovirus (BNoV) can only rely on these surrogate models to understand its potential immune responses [53,54]. These indicators can also provide some reference for evaluating the immune efficacy of a BNoV vaccine.

## 5. Conclusions

In conclusion, we successfully generated VLPs that mimic the native structure of BNoV. These VLPs are capable of eliciting specific humoral and cellular immune responses in mice, demonstrating their immunogenic potential to prevent BNoV infection. This study lays a solid foundation for the development of a BNoV vaccine and serves as a valuable reference for the subsequent creation of innovative BNoV vaccines. However, further investigation is warranted to determine the optimal immunization regimen, assess safety profiles, and evaluate protective efficacy.

## Data Availability

No new data were created in this study. The data analyzed in this study are available from GenBank.
